# Continent ileocaecocystoplasty bladder augmentation following a failed appendicovesicostomy on pediatric bladder neck stricture due to pelvic fracture urethral injury: A case report

**DOI:** 10.1016/j.eucr.2022.102288

**Published:** 2022-11-23

**Authors:** Abraham Gita Ramanda Christanto, Nanda Daniswara, Rudiyuwono Raharjo, Ardy Santosa, Eriawan Agung Nugroho, Sofyan Rais Addin

**Affiliations:** a Department of Surgery, Faculty of Medicine Universitas Diponegoro, Dr. Kariadi General Hospital, Semarang, Indonesia; b Urology Division, Department of Surgery, Faculty of Medicine Universitas Diponegoro, Dr. Kariadi General Hospital, Semarang, Indonesia; c Pediatric Surgery Division, Department of Surgery, Faculty of Medicine Universitas Diponegoro, Dr. Kariadi General Hospital, Semarang, Indonesia

**Keywords:** Bladder neck obstruction, Urinary diversion, Continent urinary reservoirs, Pelvic fracture urethral injury

## Abstract

Pelvic fracture urethral injury (PFUI) in pediatrics is rare, may involve the bladder neck, and may lead to obstruction and urinary incontinence as a lifelong disability. A 9-year-old female patient had a bladder neck injury related to PFUI after an accident when she was 6 years old and had urinary incontinence. In the previous hospital, the patient underwent appendicovesicostomy but the surgery failed. Continent ileocaecocystoplasty for a definitive treatment was done in our hospital involving the caecum, ileocaecal valve, and ileum. This procedure was delivered safely and brought a good result to the patient with no significant complications.

## 1 Introduction

Pelvic fracture urethral injury (PFUI) in pediatrics is considered rare with 2.4–7.5% incidence.^1^ Pelvic fracture indicates a high-impact trauma that can cause bladder and urethral injuries leading to urinary obstruction and also urinary incontinence as a lifelong disability.^1^ Stricture management should be individualized, depending on stricture length, bladder neck integrity, and the patient's preferences. Hosseini et al. mentioned that urinary diversion is a treatment of choice in patients with urethral defects which cannot be managed by urethroplasty.^2^ Definitive treatment should be performed to achieve continence and patient comfort.

In the continent urinary diversion, there is a need for manual dexterity of the patient/caregiver for practicing Clean Intermittent Catheterization (CIC). There are several techniques for continent urinary diversion such as Mitrofanoff appendicovesicostomy (APV), Monti procedure, continent catheterizable vesicostomy, button vesicostomy, Indiana pouch, augmentation cystoplasty, ureterosigmoidectomy, etc. In 1992, Sarosdy reported a technique of ileocaecocystoplasty with continent catheterizable stoma modified from Indiana reservoir technique.^3^ In the current report, we used a modification of the Indiana pouch continent technique using the ileocaecal segment for bladder augmentation (ileocaecocystoplasty) as a treatment for bladder neck and urethral injury after a failed APV.

## 2 Case presentation

**Fig. 1:**
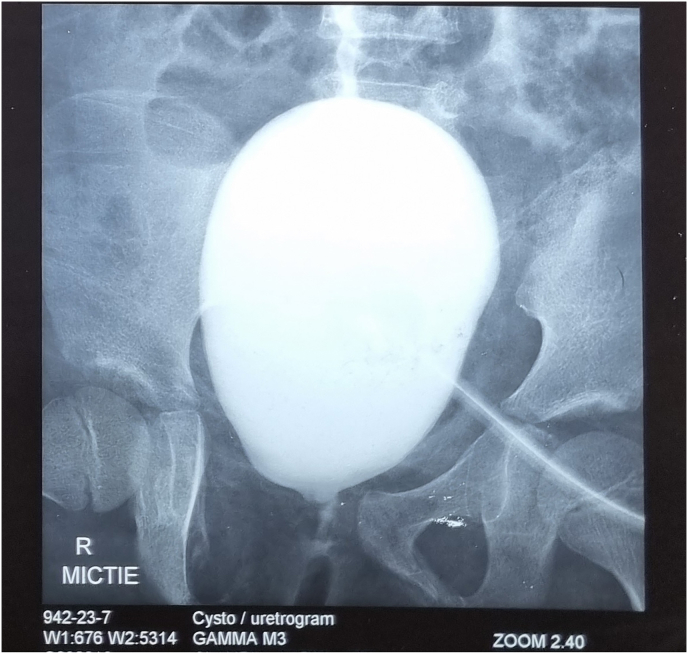
The preoperative cystourethrogram showed that the contrast (white) did not flow to the urethra indicating bladder neck obstruction and urethral stricture.

A 9-year-old female patient was in an accident three years ago, had a pubic fracture, and experienced urinary incontinence. The patient had grade V (American Association of the Surgery of Trauma/AAST scale) bladder injury which involves the bladder neck and urethra. At that time, the patient underwent continent APV at another hospital but the surgery failed and the patient maintained the use of cystostomy for urination. The bipolar cystourethrogram showed bladder neck obstruction and urethral stricture image (Fig. 1). Monti's procedure using ileal segment as a catheterizable channel and cystoplasty were considered for the second surgery. However, after discussing the risks and benefits with the family, ileocaecocystoplasty procedure as a definitive treatment was chosen.

**Fig. 2:**
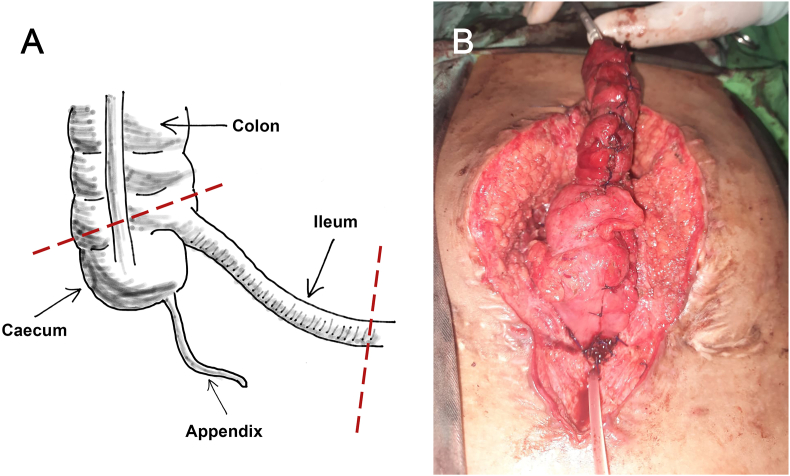
The ileocaecal segment used in ileocaecocystoplasty in this case. **(A)** The illustration of the ileocaecal segment used in this procedure, except the appendix part (marked by red dotted lines). **(B)** Intraoperative image: the bladder was anastomosed with the ileocaecal segment. (For interpretation of the references to colour in this figure legend, the reader is referred to the Web version of this article.)

**Fig. 3:**
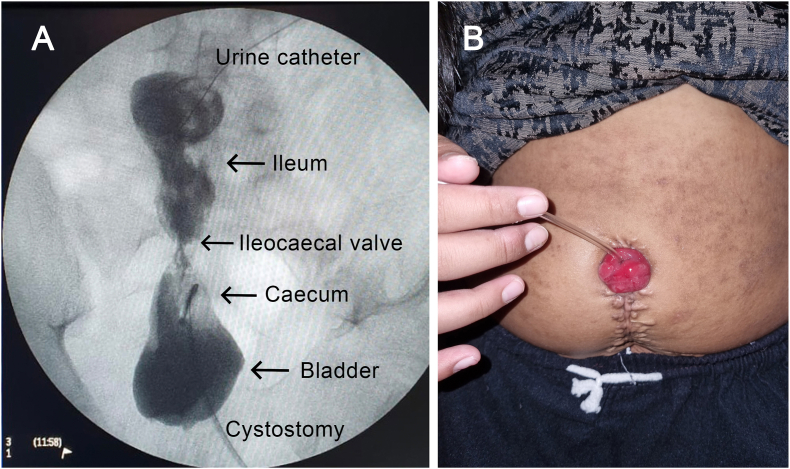
The follow-up images of the patient. **(A)** One month postoperative cystourethrogram showed that the contrast (black) flowed smoothly to fill the ileum and the bladder with no extravasation contrast on the surrounding structure. **(B)** One-year follow-up image of the patient performed self-catheterization.

On the operation, adhesion of the necrotic appendix from the previous APV was found. The appendix was removed and ileocaecocystoplasty was performed. We used 5 cm of the caecum as bladder augmentation, ileocaecal valve, and 15 cm of ileum as the efferent limb (Fig. 2A). The ileocaecal segment was resected and detubularized. The ileocolic anastomosis was done to re-establish continuity of the intestinal tract. The proximal end of the caecum was connected to the native bladder while the distal end of the ileum became an efferent limb for a stoma to the umbilicus (Fig. 2B). The patient was trained to manually input a catheter to the umbilical stoma every 4–6 hours to drain the urine from the reservoir (CIC practice). The first-month cystourethrogram showed that the contrast flowed well to the ileum and the augmented bladder (Fig. 3A). The patient did not have any complaints or experience any concerning complications (e.g. pain, incontinence, frequent diarrhea) after one year postoperative (Fig. 3B).

## 3 Discussion

Appendicovesicotomy was usually chosen as the first treatment of choice as it does not involve the bowel. In this patient, the APV failed in which the appendix was nonvascularized well and necrotic. Monti's technique with ileal segment and cystoplasty with ileocaecal segment were considered for the second surgery. But as Monti's technique complications are similar to APV, ileocaecocystoplasty procedure was preferred. Sarosdy also stated that APV had a 50% success rate and that augmentation may be required at a later time in the failure case.^3^

In the current report, continence was aimed as the objective of the procedure to improve quality of life. The bladder was preserved and was augmented with the detubularized ileocaecal segment. Although the aim was not primarily to enlarge the bladder, the patient could benefit from a slightly enlarged bladder (providing lower pressure reservoir and protecting upper urinary tract).^3^

The 9-year-old patient performs CIC well every 4–6 hours using French catheters and experiences continence. Based on Hansen et al., the short-term complication of bladder augmentation is anastomotic leak while long-term complications are bladder stones, bladder perforation, urinary tract infections, and metabolic disorders.^4^ The patient did not experience short-term complications and no long-term complications were found so far during a year evaluation.

Metabolic changes related to the use of ileocaecal segment should be anticipated. The terminal ileum plays an important role in bile salt and fat absorption, as well as vitamin B12.^5^ Compromises in this role may cause malabsorption, steatorrhea, and diarrhea. Three to five years amount of vitamin B12 is stored in the body so that the changes caused by vitamin B12 malabsorption will occur after several years.^5^ Although the patient has not experienced any complications such as frequent or suspicious occurrence of diarrhea since the surgery, long-term follow-ups and preventive measures should be taken. Renal function tests (serum creatinine and renal ultrasound) and other metabolic laboratory tests should be taken for monitoring.

## 4 Conclusion

Ileocaecocysptoplasty procedure is less risky and less extensive than a complete bladder replacement and is a recommended alternative after previous failed surgery. Continence is achieved by using the ileocaecal valve. The continent urinary diversion avoids the need for urostomy bags and plays a role in improving the patient's quality of life through good CIC practice. However, the patient still has to be observed to prevent renal function deterioration or to detect whether any metabolic changes occur.

## Declaration of competing interest

None.
